# Managing N Inputs and the Effect on N Losses Following Excretion in Open-Dirt Feedlots in Nebraska

**DOI:** 10.1100/tsw.2001.363

**Published:** 2001-12-19

**Authors:** Galen E. Erickson, Terry J. Klopfenstein

**Affiliations:** Department of Animal Science, University of Nebraska, Lincoln, USA

**Keywords:** nitrogen, cattle feedlots, nutrient management, protein requirement, volatilization

## Abstract

Nutrition will play an important role in meeting the environmental challenges of beef cattle feedlots. Nutritionists are continually refining protein requirements, and have recently adopted a new metabolizable protein (MP) system to more efficiently use nitrogen (N) and allow more accurate diet formulation. Protein requirements vary by animal age and weight during the finishing period. Our hypothesis was that formulating diets with the MP system would decrease N inputs and lead to decreased excretion and losses. Comparing industry average diets (13.5% crude protein) to phase-fed diets formulated to not exceed MP requirements decreased N inputs by 10 to 20% for calves and yearlings without affecting average daily gain. Decreasing inputs led to a concomitant decrease in N excretion (12 to 21%) and losses (15 to 33%) in open-dirt feedlot pens. N losses are variable with time of year, with averages of 60 to 70% of excreted N lost during the summer months and 40% lost during the November to May feeding periods. Protein requirements are being refined continually as more research data are collected. However, formulation to meet protein requirements, but not exceed them, is an important nutritional management option for feedlots to become sustainable.

## INTRODUCTION

Dietary manipulation is becoming an increasingly important aspect of managing waste in current confinement operations. Decreasing dietary protein in dairy[1,2] and swine operations[3] has been shown to decrease nitrogen (N) losses via volatilization. In 1996, the National Research Council (NRC) adopted the metabolizable protein (MP) system, first proposed in 1974[4], to evaluate protein requirements of beef cattle. Utilizing this system, as well as the NRC computer model, should allow nutritionists to more accurately define requirements and decrease protein supplementation without adversely affecting animal performance. From an environmental perspective, decreasing total N inputs would be advantageous to improve the sustainability of the feedlot industry.

Because primary losses of N in feedlots occur through volatilization[5], and given the impact dietary protein has on volatilization in other livestock facilities, our objective was to determine the impact of decreasing dietary protein on N and organic matter (OM) mass balance in the feedlot. Our hypothesis was that decreasing dietary protein would decrease N losses while maintaining performance if diets were formulated on a MP basis[6].

## EXPERIMENTAL METHODS

Four experiments were conducted, two each with 96 yearling crossbred steers (initial body weight [BW] = 316 ± 25 kg) fed through the summer months and two each with 96 crossbred calves (initial BW = 275 ± 32 kg) fed through the winter-spring months. Steers were assigned randomly (8 steers per pen) to either the control (referred to here as CON), or the experimental treatment (referred to as PHASE). Yearlings were fed for an average of 137 days from May to October and implanted twice with Revalor-S, with the second implant approximately 70 days from slaughter.

Yearlings were stepped-up on energy in 21 days with four diets containing 45, 35, 25, and 15% alfalfa hay, which were fed for 3, 4, 7, and 7 days, respectively. The control diet (Table 1) was formulated to provide 13.5% crude protein and 0.35% phosphorus (P) with all supplemental protein from urea. The control diet was considered typical for this region, based on published surveys[7,8]. PHASE was formulated using the 1996 NRC model and the MP system (Fig. 1) to not exceed degradable intake protein (DIP) and undegradable intake protein (UIP) requirements[6].

The difference between DIP and UIP is in the amount of protein that is utilized by rumen microbes. Since protein from high-moisture corn (HMC) is lower in UIP, and the requirement for UIP is also lower for yearlings, dry-rolled corn (DRC) was used in CON whereas the PHASE contained HMC to minimize

**TABLE 1**
**Diet Composition (% of DM) for Yearlings and Calves**

| | Yearlings | | | | Calves | | | | | | | | |
|---|---|---|---|---|---|---|---|---|---|---|---|---|---|
| | CON[a] | PHASE[a] | | | CON[a] | PHASE[a] | | | | | | | |
| Item[b] | | 1 | 2 | 3 | | 1 | 2 | 3 | 4 | 5 | 6 | 7 | 8 |
| DRC | 81.3 | | | | 82.5 | 82.5 | 82.5 | 82.5 | 82.5 | 59.5 | 35.0 | 4.5 | |
| HMC | | 67.4 | 64.6 | 61.4 | | | | | | 16.5 | 36.5 | 61.0 | 57.5 |
| C.bran | | 17.2 | 19.9 | 23.1 | | | | | | 6.5 | 11.0 | 17.0 | 25.0 |
| Liq-32 | 6.2 | | | | 5.0 | | | | | | | | |
| Molasses | | | | | | 5.0 | 5.0 | 5.0 | 5.0 | 5.0 | 5.0 | 5.0 | 5.0 |
| Fat | | 3.0 | 3.0 | 3.0 | | | | | | | | | |
| Urea | .52 | .76 | .76 | .76 | .29 | .83 | .88 | .91 | .96 | .87 | .75 | .62 | .60 |
| FM | | | | | 1.40 | 1.60 | 1.15 | .75 | .18 | | | | |
| BM | | | | | .18 | .20 | .14 | .10 | .02 | | | | |
| *CP (%)* | *13.6* | *11.2* | *11.9* | *11.5* | *13.4* | *12.7* | *12.4* | *12.1* | *11.7* | *11.5* | *11.2* | *10.8* | *10.9* |
| *UIP (%)*[c] | *4.48* | *avg.=3.67* | | | *5.16* | *5.51* | *5.23* | *4.99* | *4.64* | *4.11* | *3.68* | *3.11* | *3.02* |

[a]CON is control and PHASE is phase-fed treatments,
[b]DRC is dry-rolled corn, HMC is high-moisture corn, C. bran is corn bran, Liq-32 is a molasses based supplement containing urea.
[c]The PHASE finishers for yearling experiments and PHASE 8 finisher for calf experiments unavoidably contained more UIP than required.

*Note*: All diets contained 7.5% alfalfa and 5% supplement providing urea, feather meal (FM), and blood meal (BM) that replaced the supplement carrier, fine-ground corn.

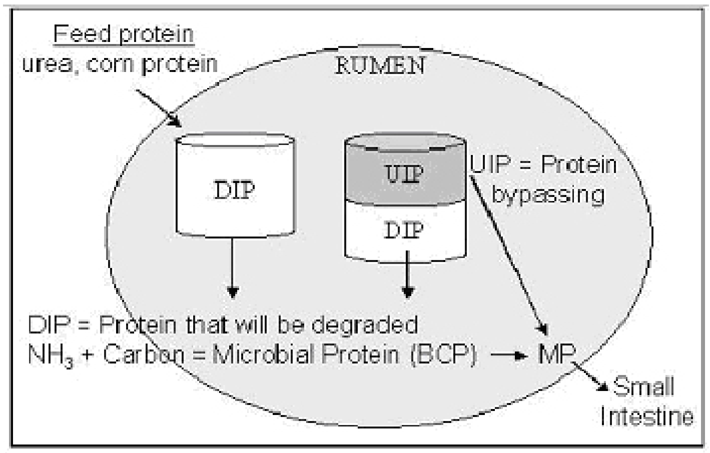

**FIGURE 1**. Diagrammatic representation of the metabolizable protein (MP) system adopted in the 1996 NRC illustrating flow of degradable intake protein (DIP) and undegradable intake protein (UIP) through the rumen. All feed proteins will be degraded by varying degrees by rumen microbes. An example is urea which is completely degradable while most other feedstuffs contain both UIP and DIP fractions. MP is the sum of microbial protein and UIP that enters the small intestine.

overfeeding of UIP. Likewise, since both DRC and HMC contain 0.25 to 0.30% P, and the requirement is 0.23% P, the PHASE treatment also contained corn bran (0.10% P) to meet, but not exceed, the P requirement predicted by the NRC model. The P balance data were previously published[9].

In the two calf experiments, calves were fed for an average of 192 days from November to May. Calves were implanted twice with Revalor-S with the second implant approximately 85 days from slaughter. Cattle were adapted to finisher diets (7.5% alfalfa) similar to the yearling experiments except that each diet was fed for 7 days. CON was formulated to provide 0.35% P and 13.5% crude protein; however, supplemental crude protein was from urea, 1.4% feather meal, and 0.18% blood meal on a dry-matter (DM) basis to provide UIP throughout the 192 days. PHASE was formulated using the 1996 NRC model to meet changing calf requirements. The first seven PHASE diets were fed for 14 days each and PHASE 8 was fed until slaughter. Since calves initially require more UIP as a percentage of total protein fed, DRC was used and gradually switched over to HMC by PHASE 7. Likewise, the P requirement also decreases with increasing weight of the animal so DRC and HMC were gradually replaced with corn bran to prevent overfeeding of P. Likewise, the P requirement also decreases with increasing weight of the animal so DRC and HMC were gradually replaced with corn bran to prevent overfeeding of P. Similar to the yearling experiments, the P balance data were previously presented[9].

Steers were fed in 12 open-dirt pens at the University of Nebraska research feedlot on the Agricultural Development and Research Center near Mead, NE. Pens were similar to experimental units used previously[10]. Animals were fed in those pens for an average of 132 days over the summer, or 183 days over the winter-spring, after which they were cleaned. Pens were cleaned in an attempt to remove all manure and minimize manure concentration in the soil. Manure was piled on the cement apron and sampled while loading. Manure was weighed wet to calculate nutrient measurements of DM, OM, and N removed in manure. Soil cores (16 locations on a grid) from the cleaned open dirt pens were sampled (0 to 15 cm) with a probe before each experiment to estimate nutrient concentration on the pen surface. Pen soil cores were collected after cleaning each pen to correct for inevitable cleaning variation.

Six runoff collection basins were constructed from soil below the pens. Following precipitative runoff events, runoff was drained, sampled, and volume measured with an ISCO 1430 air bubble flow meter (ISCO, Inc., Lincoln, NE). Due to pen design, two pens drained into one pond; therefore dietary treatments were assigned in blocks of two pens. Manure samples were freeze dried for DM to conserve N, and soil cores, feeds, and feed refusals were oven dried at 60ºC for 48 h and analyzed for N on a N analyzer (Perkin Elmer, Norwalk, CT). Runoff was analyzed wet by Kjeldahl N procedure[11]. All samples including feed ingredients, soil cores, manure, runoff, and feed refusals were analyzed for OM by ashing at 600ºC for 5 h[11].

N and OM intakes were calculated as concentration in diet * DM offered – the nutrient in feed refused * DM refused. N retention was calculated using the net protein gain equation[6]. N excretion was calculated as intake – retention. OM excretion was calculated by OM indigestibility of each diet[9], where CON diet was 20.7% indigestible and PHASE was 29.9% indigestible across all phases for yearling experiments. For the calf experiments, OM indigestibility averaged 26.2% across all phases for PHASE. Losses of N and OM were calculated as nutrient excretion – nutrient removed in manure, nutrient removed in runoff, and nutrient on the pen surface as determined by soil cores.

## RESULTS AND DISCUSSION

Gains and carcass characteristics were unaffected by dietary treatment in both calf (Table 2) and both yearling (Table 3) experiments suggesting that dietary protein was sufficient in the PHASE treatment. Yearlings gained 1.83 kg/day and calves gained 1.56 kg/day. On average, protein retained by the animal was 170.5 g/day for yearlings and 156.8 g/day for calves across both treatments and was not different between treatments[6]. If protein is 16% N (divide by 6.25), then 3.6 kg of N was retained by yearlings, and 4.6 kg by calves for the 132 and 183 days in the pens, respectively. Subtle differences were observed in feed efficiency, which presumably is related to grain source differences between treatments.

N intake, expressed as total kg per head, was reduced by feeding the PHASE in yearling (6.1 kg) and calf (4.2 kg) experiments (Table 4). N retention in the animal was unaffected since gains were unaffected by treatment. Therefore, feeding PHASE reduced N excretion by 6.1 kg (46 g/day) in the yearling experiments and 4.2 kg (23 g/day) in the calf experiments.

Soil core values suggest that cleaning differences did occur between treatments for the summer yearling experiments. When expressed as total kg, N removed in manure when adjusted for

**TABLE 2**
**Performance of Calves Fed either Conventional Protein and Phosphorus Levels (CON) or Phase-Fed Diets (PHASE) Formulated to not Exceed Requirements (Due to Year by Treatment Interaction for Feed Efficiency, Means are Separated by Treatment and Year)**

| | Year 1 | | | Year 2 | | | | yr × trt |
|---|---|---|---|---|---|---|---|---|
| Item | CON | PHASE | P | CON | PHASE | P | SEM | P |
| Initial weight, kg | 245 | 246 | NS | 304 | 306 | NS | 1 | NS |
| Final weight, kg | 563 | 567 | NS | 585 | 575 | NS | 5 | NS |
| DMI, kg/d | 9.3 | 9.3 | NS | 9.1 | 9.5 | 0.05 | 0.1 | 0.11 |
| ADG, kg/d | 1.65 | 1.66 | NS | 1.48 | 1.43 | 0.16 | 0.03 | 0.23 |
| ADG/DMI | 0.177 | 0.179 | NS | 0.163 | 0.150 | 0.01 | 0.002 | 0.01 |

**TABLE 3**
**Performance of Yearlings Fed either Conventional Protein (CON) or Phase-Fed Diets (PHASE) Formulated to not Exceed Requirements (Due to No Year by Treatment Interaction [$p > 0.10$], Means were Combined Across Years)**

| Item | CON | PHASE | SEM | trt effect *P* |
|---|---|---|---|---|
| Initial weight, kg | 315 | 316 | 1 | NS |
| Final weight, kg | 563 | 570 | 3 | NS |
| DMI, kg/d | 11.4 | 11.1 | 0.1 | 0.03 |
| ADG, kg/d | 1.81 | 1.85 | 0.02 | NS |
| ADG/DMI. kg gain/kg feed | 0.158 | 0.166 | 0.002 | 0.01 |

**TABLE 4**
**N Balance in the Feedlot for the Yearling and Calf Experiments Separated by Dietary Treatment (All Values Expressed as kg per Steer over the Entire Feeding Period)**

| | Yearlings (132 d) | | | | Calves (183 d) | | | |
|---|---|---|---|---|---|---|---|---|
| Item | CON | PHASE | SEM | P= | CON | PHASE | SEM | P= |
| Intake | 33.1 | 27.0 | 0.3 | 0.01 | 37.0 | 32.8 | 0.4 | 0.01 |
| Retention[a] | 3.6 | 3.6 | <.1 | 0.80 | 4.6 | 4.6 | <.1 | 0.28 |
| Excretion[b] | 29.5 | 23.4 | 0.3 | 0.01 | 32.4 | 28.2 | 0.4 | 0.01 |
| Manure | 5.9 | 8.9 | 0.3 | 0.01 | 19.8 | 18.9 | 1.2 | 0.60 |
| Soil[c] | 1.7 | -.4 | 0.6 | 0.03 | -1.7 | -2.9 | 0.8 | 0.28 |
| Runoff | 1.0 | .7 | 0.1 | 0.07 | 1.0 | 1.0 | 0.1 | 0.74 |
| Volatilized[d] | 20.9 | 14.2 | 0.7 | 0.01 | 13.3 | 11.3 | 1.4 | 0.32 |
| % volatilized | 70.9 | 60.7 | | | 41.1 | 40.1 | | |
| Manure+soil[e] | 7.6 | 8.5 | 0.7 | 0.39 | 18.1 | 15.9 | 1.3 | 0.24 |

[a]N retention based on ADG, NRC equation for retained energy and retained protein.
[b]N excretion calculated as intake minus retention.
[c]Soil is core balance on pen surface before and after each experiment; negative values suggest removal of nutrient present before initiation of experiment.
[d]Volatilized calculated as excretion minus manure minus soil minus runoff.
[e]Manure+soil corrects what was hauled at cleaning by soil nutrients remaining or removed from pen surface when compared with nutrients on the pen surface before the experiment.

pen soil N was not different between dietary treatments for both yearling and both calf experiments. Interestingly, decreasing N inputs to feedlot cattle did not influence N hauled in manure, thereby maintaining fertilizer value. When expressed as percentage of N excreted, 26 and 36% of N was available for hauling in the CON and PHASE treatments, respectively, in the yearling experiments. For the calf experiments, PHASE numerically decreased N in manure. As a percentage of N excreted, 56% was hauled in manure. The percentages expressed here for manure N are related to the N in manure + soil. Manure should be corrected for N either left on the pen surface or N taken from pen soil. Therefore, all percentages are corrected for the nutrient balance of the soil cores taken before and after each experiment.

Nutrient balance for the calf experiments was more variable than for the summer-feeding, yearling experiments. Due to greater MP requirements for calves, N intake was not reduced to the same degree with the calf experiments when compared with the yearling experiments (4.2 vs. 6.1 kg). Both the variability as well as the smaller difference in N excretion between treatments led to difficulty in determining significant differences in N mass balance for the calf experiments compared to yearling experiments.

OM excretion was increased by feeding PHASE (Table 5) because corn bran, which is less digestible than corn grain, replaced part of the dietary corn. Overall, OM balance was similar to N balance, except that very little OM (probably not different from zero) was volatilized from pens during the winter calf experiments. N and OM leaving the feedlot via surface runoff did not account for large portions of total nutrient excreted (<3.5 and <7% for N and OM, respectively). The runoff percentages are in agreement with the 3to 6% losses reported previously[12] and agree with percentages using similar dietary treatments[10].

For the yearling experiments, the majority of N losses from feedlot pens are presumably via volatilization as ammonia and other N-containing gases during the summer, with estimates of 70.9 and 60.7% for CON and PHASE, respectively. These percentages are greater than the previous estimates[5,12], but agree with previous experiments utilizing similar procedures[10]. When expressed as total N over the entire feeding period, PHASE

**TABLE 5**
**OM Balance in the Feedlot for the Yearling and Calf Experiments Separated by Dietary Treatment (All Values Expressed as kg per Steer over the Entire Feeding Period)**

| | Yearlings (132 d) | | | | Calves (183 d) | | | |
|---|---|---|---|---|---|---|---|---|
| Item | CON | PHASE | SE | P= | CON | PHASE | SE | P= |
| Intake | 1463 | 1399 | 12 | 0.01 | 1508 | 1595 | 17 | 0.01 |
| Excreted[a] | 302 | 418 | 3 | 0.01 | 339 | 425 | 4 | 0.01 |
| Manure | 110 | 180 | 6 | 0.01 | 367 | 396 | 24 | 0.38 |
| Soil[b] | 19 | -34 | 14 | 0.02 | -34 | -39 | 17 | 0.83 |
| Runoff | 20 | 15 | 2 | 0.08 | 8 | 13 | 1 | 0.02 |
| Volatilized[c] | 153 | 257 | 16 | 0.01 | -2 | 55 | 26 | 0.13 |
| Manure+soil[d] | 129 | 146 | 15 | 0.43 | 333 | 358 | 25 | 0.50 |

[a]OM excretion calculated from digestibility data from corn and corn bran diets.
[b]Soil is core balance on pen surface before and after each experiment; negative values suggest removal of nutrient present before the experiment.
[c]Volatilized calculated as excretion minus manure minus soil minus runoff.
[d]Manure+soil corrects what was hauled at cleaning by soil nutrients remaining or removed from pen surface.

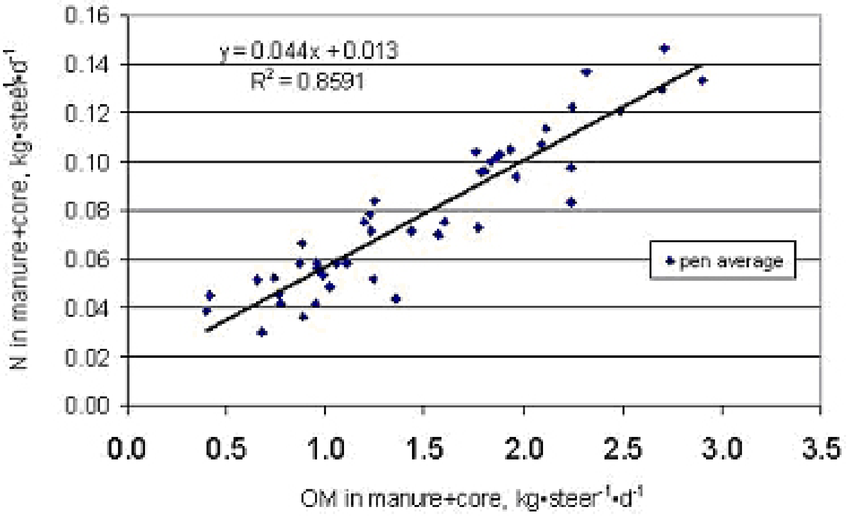

**FIGURE 2**. Regression of manure OM and manure N adjusted for soil for all four experiments where each dot represents a pen (48). N excretion varied from 0.15 to 0.22 kg per steer per day.

resulted in 14.2 vs. 20.9 kg per steer lost for CON. The 32% decrease can be primarily attributed to the decrease in N excreted by the steers and some improvement in N captured in manure. For the calf experiments, N losses were 40.5% of excreted N for both treatments during the winter and N loss was numerically lower (11 vs. 13 kg) for PHASE compared to CON treatment for calves.

Dietary N and indigestibility of dietary OM rather than ingredient composition, runoff amounts, and variation in pen cleaning may be the largest factors influencing manure characteristics. Considerable differences from year 1 to year 2 were observed in all measurements for nutrient balance except the manure data. Averaged across both treatments, N in manure for the summer experiments was 7.3 and 7.4 ± 0.33 kg per steer for years 1 and 2, respectively. OM in manure was 147 and 143 ± 6.4 kg per steer for the summer experiments for years 1 and 2, respectively. Likewise, N in manure from the winter-spring experiments were 18.0 and 20.6 ± 1.2 kg per steer for years 1 and 2, respectively. Indigestibility of OM influences manure OM; see Fig. 2 for an illustration of the relationship between N in manure and OM in manure for all four experiments. As OM increases, N removed in manure increases according to the following regression equation: N = 0.044 * OM + 0.013 where N and OM are expressed as kg per steer per day and an $R^2 = 0.86$. This relationship is similar to previous summer feeding experiments where source and amount of dietary fiber were evaluated[10]. Likewise, Dewes[13] measured ammonia release from cattle slurry and observed a decrease in ammonia production when more litter (OM) was added to slurry.

If two diets differ in protein content, then the higher-protein diet will lead to increased urinary N excretion as urea[14]. The reason is that excess protein above the requirement must be metabolized to urea and excreted in urine. In these experiments, similar fecal N was probably excreted between treatments, but less urea N was excreted in urine by cattle on the PHASE treatment due to lower protein intake. In general, the urine is the predominant contributor to volatilization when feces and urine are compared[15]. Because urea-N is rapidly converted to ammonia due to prevalence of urease enzyme[16], then presumably more N would be volatilized if protein is overfed.

Based on current feeding trends in Nebraska and the U.S., approximately 64 to 70% of cattle are fed from November to May[17]. Only 30 to 36% of cattle are in the feedlot from June to October for 1995 to 1998. If yearly volatilization rates are based on weighted averages for cattle on feed (65:35 ratio), then the amount of N that volatilizes is 53.5% of N excreted for the CON treatment. Similar calculations for PHASE result in 48.2% of N excreted being volatilized. The percentages are not greatly different, but total kg of N volatilized was reduced by 25% when PHASE was fed compared with CON. Accounting for cattle inventory when calculating losses should be more accurate than basing N losses on summer and winter finishing systems separately. However, in these experiments, winter and summer finishing characteristics are confounded by type of animal (calf vs. yearling) because each has unique feeding characteristics. Despite estimates of 60 to 70% of N volatilizing in the summer experiments, the yearly estimates accounting for cattle inventory trends are more similar to previous estimates[5,12].

## CONCLUSIONS

Feeding less protein decreased N losses without compromising performance. Presumably, lower protein intake decreased N excretion via urine, thereby decreasing concentrations of ammonium on the pen surface. Because of the concerns with N losses to the atmosphere, cattle feedlot operators should minimize excess dietary protein.

| | | |
|---|---|---|
| Received: | July | 28, 2001 |
| Revised: | November | 6, 2001 |
| Accepted: | November | 7, 2001 |
| Published: | December | 19, 2001 |
